# Prospective memory impairments in heavy social drinkers are partially overcome by future event simulation

**DOI:** 10.1007/s00213-015-4145-1

**Published:** 2015-11-27

**Authors:** Bradley Platt, Sunjeev K Kamboj, Tommaso Italiano, Peter G Rendell, H Valerie Curran

**Affiliations:** Clinical Psychopharmacology Unit, University College London, Gower Street, London, WC1E 6BT UK; Cognition and Emotion Research Centre, Australian Catholic University, Melbourne, Australia

**Keywords:** Prospective memory, Alcohol, Alcohol use disorders, Future event simulation

## Abstract

**Background:**

Recent research suggests that alcohol acutely impairs prospective memory (PM), and this impairment can be overcome using a strategy called ‘future event simulation’ (FES). Impairment in event-based PM found in detoxifying alcohol-dependent participants is reversed through FES. However, the impact of the most common problematic drinking patterns that do not involve alcohol dependence on PM remains unclear.

**Aims:**

Here, we examine the impact of frequent heavy drinking on PM and the degree to which any impairments can be reversed through FES.

**Methods:**

PM was assessed in 19 heavy drinkers (AUDIT scores ≥15) and 18 matched control participants (AUDIT scores ≤7) using the ‘Virtual Week’ task both at baseline and again following FES.

**Results:**

Heavy drinkers performed significantly worse than controls on regular and irregular time-based PM tasks. FES improved the performance of controls but not of heavy drinkers on time-based tasks. In contrast, FES improved heavy drinkers’ performance on event-based PM tasks.

**Conclusions:**

These findings suggest that heavy drinkers experience deficits in strategic monitoring processing associated with time-based PM tasks which do not abate after FES. That the same strategy improves their event-based PM suggests that FES may be helpful for individuals with problematic drinking patterns in improving their prospective memory.

## Introduction

Prospective memory (PM) is the ability to enact intended actions at an appropriate moment in the future (Ellis and Freeman 2008) and can be event and/or time-based. Time-based tasks are those in which the intended action is performed at a specified time of day or after a specific amount of time has elapsed (e.g. taking medication at 8 a.m. or remembering to call back a friend in 20 min time) and relies on self-initiated behaviours. In contrast, event-based PM is promoted by an external situation or cue (e.g. remembering to pick up a prescription on your way home from work or to turn off the oven when a timer buzzes). Significant deficits in PM would have important implications for our ability to carry out basic planned actions (Rendell and Henry 2009).

Alcohol’s acute and chronic impairment of retrospective memory has been documented extensively (e.g. Bisby et al. 2010; Curran and Weingartner 2002) but only recently has attention been paid to the effects of alcohol on prospective memory. A moderate single dose of alcohol (32–40 g of ethanol) given to light social drinkers impaired both event- and time-based task-performance (Leitz et al. 2009; Paraskevaides et al. 2010). In addition, evidence has recently been found for deficits in PM in non-intoxicated people with alcohol dependence (Griffiths et al. 2012). Beyond causing difficulties in daily functioning, such impairments may be particularly relevant for those trying to abstain from alcohol use, since the ability to apply planned abstinence strategies relies crucially on intact PM (Tobias 2009). However, the extent and severity of PM impairment in people with alcohol use disorders remain poorly understood. In particular, we do not know the extent to which the time or event domains of PM (see below) are differentially affected or how impairment varies with the pattern and severity of alcohol use (e.g. dependence versus heavy daily alcohol use versus binge drinking). To date, there appear to be only two studies which have investigated PM in adults with alcohol use disorders using objective measures of PM. One examined recently detoxified people with alcohol dependence and found significant impairment on event- but not time-based tasks relative to non-dependent social drinkers (Griffiths et al. 2012). The observed level of impairment was strongly associated with the indices of alcohol use. However, a study with adult binge drinkers found that they performed significantly worse on time- but not event-based tasks than non-binge drinkers (Heffernan and O’Neill 2012). As such, the potential impairment associated with harmful drinking remains unclear.

Recent studies have focused on the use of cognitive rehabilitation to improve performance of patients with neuropsychiatric disorders through the use of compensatory strategies (Hurford et al. 2011). Such strategies may also have relevance to the treatment of cognitive deficits associated with alcohol use disorders. In the case of prospective memory, our previous set of experimental studies suggests that some acute alcohol-induced deficits can be overcome through the use of a specific cognitive rehearsal strategy—future event simulation—which involves prior rehearsal in mental imagery of to-be-performed tasks (Leitz et al. 2009; Paraskevaides et al. 2010; Griffiths et al. 2012).

Here, we examine PM in harmful, non-dependent drinkers, defined as those with scores on the Alcohol Use Disorders Identification Test (AUDIT) greater than 13 and 15 for women and men, respectively. These cut-offs were chosen to those likely to have a high level of problems according to the criteria outlined in Babor (2002). Drawing upon previous research (Griffiths et al. 2012), we predicted that heavy drinkers would perform significantly worse than healthy controls on event-based PM tasks in the Virtual Week (VW), an objective measure of PM ability. Furthermore, we hypothesised associations between PM deficits and indices of alcohol use. Finally, on the basis of our previous findings (Griffiths et al. 2012), we predicted that future event simulation would improve performance on time but not event-based PM tasks in control, non-harmful drinkers only. Given the role of attention, memory and executive function in PM processes, these domains were also assessed to examine their potential relationships to PM performance.

## Method

### Design and participants

An independent group design was used to compare heavy drinkers with control, social drinkers. Inclusion criteria for heavy drinkers were Alcohol Use Disorders Identification Test (AUDIT) scores of ≥13 and 15 for women and men, respectively; for controls, an AUDIT score of <7 was the criterion (Babor et al. 2001).

Exclusion criteria were a current or historical diagnosis of dependence on any substance; any neurological condition; a history of traumatic brain injury; current or historical experiences of psychosis; current diagnosis of a learning disability; reading difficulties; current use of anti-psychotic medication or benzodiazepines. Each control participant was selected to match a heavy drinker as closely as possible in age, gender and education.

Participants were recruited from the University College London (UCL) community and locale. They were informed that the study investigated the effects of alcohol use on thinking and were asked to complete an online survey with both demographic and substance use questionnaires to determine their eligibility to take part.

The study was approved by the University College London ethics committee, and all participants provided written, witnessed, informed consent.

### Measures

#### Prospective memory

The Virtual Week (VW; Rendell and Craik 2000) is a computer-based measure of PM abilities. Using a board game format, participants move a counter around a virtual board by rolling an electronic die. Each circuit of the board signifies a waking day. In the version of the Virtual Week used here, participants were asked to complete five circuits of the board (one practice and four experimental circuits). The virtual time of day is displayed on a 24-h clock in the centre of the board and changes with the counter’s movement around the board. When the counter lands on or passes each square on the board, seven and a half minutes elapses on the clock. On the board, there are ten event squares. When a participant’s counter lands on or passes an event square, they are asked to click an ‘event card’ button on the board which describes an activity relevant to the time of day. For example, the first event cards simulate morning activities (e.g. breakfast) and the last simulate evening activities (e.g. dinner). Overall, the board game simulates the ongoing cognitive and behavioural activity within which PM tasks are performed in daily life. Along with these virtual activities, participants are asked to make one of three actions in response to any given activity (e.g. what to have for breakfast). This ensures that participants have read and encoded the event cards’ information.

At the start of the VW, participants are presented with four ‘regular’ PM tasks that need to be completed on each virtual day (one circuit of the board). These tasks are designed to simulate regular tasks that occur in everyday life, with two of these being event-based (cued by specific activity: breakfast or dinner) and two being time-based (cued by the passing of time on the board: 11:00 a.m. and 21:00 p.m.). At the start of each virtual day, participants are presented with four ‘irregular’ PM tasks that involve one-off and non-recurring intentions. These tasks are designed to simulate more occasional tasks that occur in everyday life, again with two of these being event-based and two being time-based.

One trial day was used to orientate participants to the task and give them practice on it. Participants then completed 2 days of the VW without future event simulation. Following completion of these first 2 days, participants were taught and practiced the use of future event simulation. Specifically, they were instructed to set each PM task in their own everyday life and imagine themselves doing it in as much detail as possible including the setting and time of day. They then completed another 2 days of the VW. Whenever participants were presented with an irregular PM task across these 2 days, they were prompted by the researcher to imagine performing the activity (future event simulation). For each use of future event simulation, participants were asked to rate on a five-point Likert scale the vividness of their image and their impression of living the imaged experience.

#### Memory

The story recall subtest of the Rivermead Behavioural Memory Test (RBMT: Wilson et al. 2003) was administered. Participants listen to a short passage of prose and recall it immediately and again after a 20-min delay filled by other assessments. Scoring was standard. The Wechsler (2008) Digit Span Task was used as an assessment of verbal memory span (digits forwards) and working memory (digits backwards).

#### Executive function

Verbal fluency: participants were given 60 s to generate as many words as possible that begin with the letter B. Semantic fluency: participants were given 60 s to generate as many words in a specific category (e.g. animals). Trail Making Test (TMT; Reitan and Wolfson 1994): participants completed parts A and B of this standard task, and time to complete each was recorded along with errors.

#### Attention

The Single Digit Cancellation task (SDT; White and Lintzeris 2010) is a brief paper and pencil task assessing processing speed and selective attention (Lezak 1983) with measures being time to complete and omission errors.

#### Premorbid intelligence

The Spot-the-Word test (STWT; Baddeley et al. 1993) is a brief lexical decision test that was used as an index of premorbid intelligence.

#### Mood

The Hospital Anxiety and Depression Scale (HADS; Zigmond and Snaith 1983) was used to assess mood and anxiety.

#### Alcohol and drug use

The Alcohol Use Disorders Identification Test (AUDIT) is a self-administered structured questionnaire with ten questions and was developed to detect patterns of heavy alcohol use (Babor et al. 2001). In addition, drug use was assessed with a screening questionnaire in which participants self-rated their use of illicit drugs, benzodiazepines and tobacco on a five-point Likert scale: 0 = never; 1 = less than monthly; 2 = monthly; 3 = weekly; 4 = daily or almost daily.

### Procedure

Participants were asked to abstain from alcohol and all recreational drugs for 24 h before testing. They were breathalysed using a Lion 500 portable Alcometer (Lion Instruments, UK) upon arrival to ensure a reading of 0.00 prior to testing. All participants produced this reading. The order of tasks was as follows: Virtual Week; immediate story recall from the RBMT; Digit Cancellation Task; Spot-the-Word test; verbal and semantic fluency tests; forward and backwards digit span; Trails Making Test; delayed story recall from the RBMT. Participants were compensated (£6/h) for their time. Test administration lasted between 60 and 90 min.

### Data analysis

Performance on the VW was scored as early (target item was performed before the correct time criterion), correct (target item was performed at the correct time or before the next roll of the dice) or late (target item performed after the correct time criterion but before the end of the virtual day). Missed responses (failure to remember target item) were also recorded.

Where assumptions of homogeneity of variance and univariate normality could not be made and transformations had no effect, non-parametric statistical methods were used.

All VW data were analysed with a 2 × 2 × 2 × 2 repeated measures ANOVA with task regularity (irregular versus regular), task type (event-based versus time-based) and pre- and post-future event simulation as within-subjects factors, and group (heavy drinkers versus controls) as a between subject factor. To explore interactions, we used post hoc Bonferroni adjusted pairwise comparisons. Relationships between interaction effects and other variables (e.g. alcohol use) were explored with non-parametric correlations. For significant results, effect sizes were calculated using Hedges *g* with effect sizes between 0.20 and 0.49 being defined as small, between 0.50 and 0.79 defined as medium and greater than 0.80 defined as large (Cohen 1988). Outliers were defined as having values ≥3 SDs from the mean of the variable and were excluded.

## Results

### Demographics

The groups were well matched in terms of gender (heavy drinker group: 8 women; 11 men; control group: 5 women; 13 men). The heavy drinkers were significantly younger (*M*(SE) = 25.55 (2.36)) than controls (*M*(SE) = 27.60 (1.59)) (*U* = 93.00, *p* = 0.02). As expected, heavy drinkers had higher scores on the AUDIT (*M*(SE) = 22.60 (0.90)) compared to controls (*M*(SE) = 3.75 (0.42)) (*U* = 29.00, *p* < 0.001) and reported a greater number of drinking days per week (*M*(SE) = 4.65 (0.30)) than controls (*M*(SE) = 1.60 (0.34)) (*U* = 29.00, *p* < 0.001). Groups did not differ significantly in premorbid (STW) scores, anxiety or depression (all *p* values >0.05).

### Substance use

As shown in Table 1, more people in the heavy drinking group had ever used cannabis (*χ*^2^_4_ = 13.65, *p* = 0.009), cocaine (*χ*^2^_3_ = 8.12, *p* = 0.042), MDMA (*χ*^2^_3_ = 9.67, *p* = 0.022) and cigarettes (*χ*^2^_4_ = 14.48, *p* = 0.006). However, frequency of use of illicit drugs was low (between once a month and less than once per month.

**Table 1:** Number of participants reporting ever using ‘other’ drugs and mean (SE) frequency of use in the heavy drinker and control group

	Heavy drinker group		Control group	
	*n*	*M* (SE)	*n*	*M* (SE)
Cannabis	12	1.67 (0.28)	1	1.00 (0.00)
Cocaine	7	1.71 (0.29)	0	0.00 (0.00)
MDMA	8	1.50 (0.27)	0	0.00 (0.00)
Benzodiazepine	5	1.00 (0.00)	0	0.00 (0.00)
Speed	3	1.00 (0.00)	0	0.00 (0.00)
Smoking	12	2.83 (0.34)	1	1.00 (0.00)

Note: Participants were asked to self-rate their drug use on a five-point Likert scale: 0 = never; 1 = less than monthly; 2 = monthly; 3 = weekly; 4 = daily or almost daily

### Virtual week

#### Vividness and living impression ratings

There was no difference in ‘vividness of imagery’ ratings during future event simulation (FES) between the heavy drinkers (*M*(SE) = 30.26(1.44)) and control participants (*M*(SE) = 30.12 (1.31)) (*p* > 0.05). There was also no difference in ‘impression of living’ ratings during FES between the heavy drinkers (*M*(SE) = 26.16 (1.91)) and control participants (*M*(SE) = 25.76 (1.97)) (*p* > 0.05).

#### Correct responses

**Fig. 1:**
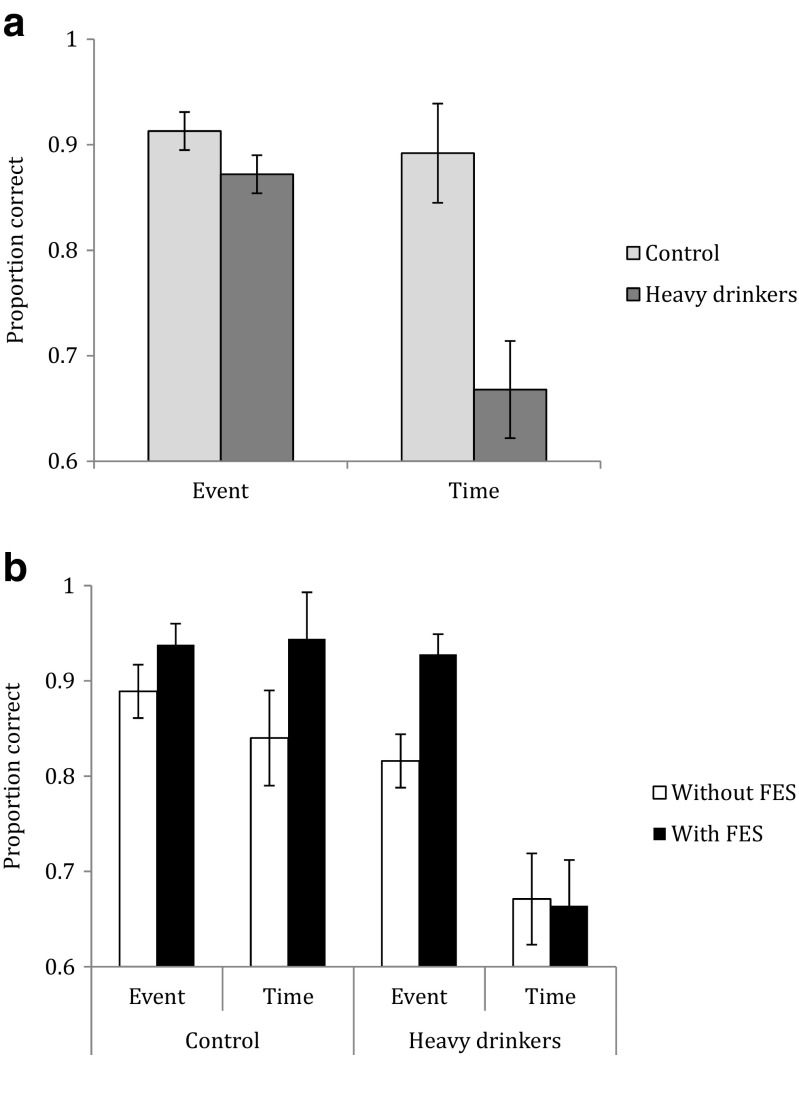
**a** Mean (SE) proportion of correct responses on event- and time-based tasks in virtual week for heavy drinkers and controls. **b** Mean (SE) proportion correct responses (summed over task regularity) for the heavy drinkers and control group as a function of task type and future event simulation

Table 2 displays mean (SE) correct responses by task regularity and type and pre- and post-future event simulation in the two groups. There were interactions between group × task type × future event simulation (*F*_1,35_ = 7.70, *p* = 0.009), group × task type (*F*_1,35_ = 10.45, *p* = 0.003) and regularity × task type (*F*_1,35_ = 5.36, *p* = 0.03). Significant main effects of group (*F*_1,35_ = 10.70, *p* = 0.002), task type (*F*_1,35_ = 15.75, *p* < 0.001) and future event simulation (*F*_1,35_ = 13.05, *p* = 0.001) were also found. Bonferroni adjusted pairwise comparisons showed the heavy drinkers had significantly fewer correct responses for time-based tasks than controls (*F*_1,35_ = 11.81, *p* = 0.002; Fig. 1).

**Table 2:** Mean (SE) proportion of correct responses for the heavy drinking group (*n* = 19) and control group (*n* = 18) by task regularity and type and future event simulation

			Future event simulation	
Group	Task regularity	Task type	Without	With
Heavy drinkers	Regular	Event	0.80 (0.04)	0.92 (0.03)
	Regular	Time	0.75 (0.07)	0.70 (0.08)
	Irregular	Event	0.83 (0.05)	0.93 (0.03)
	Irregular	Time	0.59 (0.07)	0.63 (0.07)
Control	Regular	Event	0.88 (0.04)	0.93 (0.03)
	Regular	Time	0.92 (0.04)	0.94 (0.03)
	Irregular	Event	0.90 (0.04)	0.94 (0.03)
	Irregular	Time	0.76 (0.06)	0.94 (0.03)

Bonferroni adjusted pairwise comparisons showed future event simulation improved heavy drinkers performance only on event-based tasks (*F*_1,35_ = 10.63, *p* = 0.002, 95 % CI [0.34, 1.69], *g* = 1.02) and improved control participants’ performance only on time-based tasks (*F*_1,35_ = 10.07, *p* = 0.003; Fig. 1b).

Within the heavy drinkers, there were no correlations between performance on time- or event-based tasks and any index of alcohol use including AUDIT scores and days per week of alcohol use, errors on digit cancellation or errors on the trails B test. Within the control group, there was only a negative correlation between performance on event-based tasks without future event simulation and AUDIT scores (*r*_s_ = −0.42, *p* = 0.04), although this effect would not be considered significant if an appropriate adjustment for multiple testing was applied.

#### Correct plus late responses

To further explore the nature of PM group differences, data for the correct and late responses were summed (see Table 3). With the addition of late responses, there was an interaction between group × task type (*F*_1,35_ = 7.48, *p* = 0.010) and a main effect of task type (*F*_1,35_ = 12.32, *p* = 0.001). Bonferroni adjusted pairwise comparisons showed a similar pattern of results to that obtained from analysis without late responses: the heavy drinkers had significantly fewer correct + late responses for time-based tasks than the control group (*F*_1,35_ = 5.73, *p* = 0.022), but there was no difference for event-based tasks (*F*_1,35_ = 0.47, *p* > 0.05).

**Table 3:** Mean (SE) proportion of correct plus late responses for the heavy drinking group (*n* = 19) and control group (*n* = 18) by task regularity and type and future event simulation

	Future event simulation			
Group	Task regularity	Task type	Without	With
Heavy drinkers	Regular	Event	0.97 (0.03)	0.97 (0.02)
	Regular	Time	0.80 (0.07)	0.87 (0.06)
	Irregular	Event	0.88 (0.40)	0.95 (0.02)
	Irregular	Time	0.80 (0.05)	0.86 (0.04)
Control	Regular	Event	0.97 (0.02)	0.99 (0.01)
	Regular	Time	0.96 (0.02)	0.96 (0.03)
	Irregular	Event	0.93 (0.03)	0.94 (0.03)
	Irregular	Time	0.89 (0.05)	0.97 (0.03)

### Memory and executive function

The only group differences to emerge were a greater number of errors of omission on the digit cancellation test (*U* = 66.00, *p* = 0.003), and errors on the trail B test (*U* = 119.00, *p* = 0.025) in the heavy drinking group compared to the control group (Table 4).

**Table 4:** Mean (SE) task scores of heavy drinking group (*n* = 19) and controls (*n* = 18) on neuropsychological tests

	Heavy drinkers	Control group
Memory		
Digit span forwards	7.37 (0.24)	7.89 (0.27)
Digit span backwards	5.53 (0.31)	6.39 (0.27)
Story—immediate recall	8.50 (0.55)	8.36 (0.83)
Story—delayed recall	7.34 (0.62)	7.53 (0.84)^a^
Attention		
SDC—time (s)	51.42 (2.69)^a^	59.61 (5.52)
SDC—omissions*	4.00 (1.19)^a^	1.17 (0.37)
Executive function		
Letter fluency	17.26 (0.83)	19.39 (1.29)
Category fluency	24.63 (1.06)	25.33 (1.38)
Trail A—time	20.20 (1.54)	17.53 (1.18)^a^
Trail A—errors	0.11 (0.07)	0.00 (0.00)^a^
Trail B—time	39.81 (2.81)	39.49 (3.91)^a^
Trail B—errors*	0.68 (0.39)	0.00 (0.00)^a^

*p < 0.05

^a^ *n* = 17

## Discussion

This is the first study to examine prospective memory and future event simulation in heavy drinkers. Our first main finding was that heavy drinkers perform significantly worse than control participants on time-based tasks regardless of task regularity. The heavy drinkers’ AUDIT scores ranging from 15 to 28 indicated a high level of alcohol problems; 14 participants scored 20 or more, which indicates that a clinical evaluation should be made of their possible dependence (Babor 2002). Apart from cigarette smoking in the heavy drinkers, the frequency of other drug use was very low for both groups with those who did use illicit drugs using them, on average, less than once a month. Although heavy drinkers were approximately 2 years younger than control group and made more errors on both the digit cancellation task and trail B test, there were no correlations between the performance on the VW and these variables in heavy drinkers. Thus, it is unlikely that age, other cognitive deficits or other substance use account for our prospective memory findings. Furthermore, the two groups did not differ on measures of premorbid IQ, immediate and delayed prose recall.

Our second main finding concerned the effects of future event simulation. When heavy drinkers were instructed to use this strategy at encoding, their performance on event- but not time-based tasked improved significantly. In contrast, the same simulation strategy improved the control group’s performance on time-based tasks; there was no improvement on event-based tasks as controls were at ceiling prior to FES. The improvement in time-based tasks only by controls is unlikely to reflect differences in imagery ability as there were no group differences in either vividness of imagery or impression of living ratings when using future event simulation. Furthermore, the heavy drinkers did use FES to effectively enhance their performance in event-based tasks.

Griffiths et al. (2012) used the same virtual week task with alcohol-dependent in-patients after they had completed a 7–10-day medically assisted detoxification and were no longer receiving benzodiazepines. They found that patients showed a deficit in event-based PM that was strongly correlated with measures of alcohol use. Future event stimulation made no difference to their performance on either event- or time-based tasks. These participants were older (average 42 years) than the heavy drinkers in the present study (average 25 years) and had a much longer, heavier and sustained alcohol abuse history. Another study with binge drinkers also aged around 25 years reported time-based PM impairments (Heffernan and O’Neill 2012) and suggests that time- but not event-based PM is disrupted in people with alcohol use problems that are less severe than those with a longer history of addiction. It is possible therefore that impairments in event-based PM only develop with repeated, ongoing and heavy alcohol use over years. In terms of causation, it is also possible that PM deficits pre-date and predispose to problematic alcohol use. Prospective longitudinal studies would be needed to tease apart such effects.

The control group of social drinkers in Griffiths et al.’s (2012) study showed precisely the same pattern as the social drinkers in the present study: a significant improvement in time- but not event-based prospective memory following future event stimulation. This replication strengthens our differential findings in heavy alcohol users as compared with older individuals with diagnosed alcohol dependence. It suggests an emerging alcohol-PM spectrum. Healthy young adults who drink within recommended UK guidelines (14 units per week for women; 21 for men) show significant acute alcohol-induced deficits on both time- and event-based virtual week PM tasks which were completely overcome by future event simulation (Paraskevaides et al. 2010).

We recorded not only correct responses made at the correct time but also those made later. These timing errors were particularly common with irregular time-based tasks. When correct responses regardless of timing errors were analysed in combination, heavy drinkers were still significantly impaired compared with controls. There are several possible explanations for this finding. These errors suggest that participants do not forget some PM tasks altogether but fail to associate them with a specific time for enactment. Such errors whereby you forget to enact a plan at a precise point in time are clearly common experiences in daily life.

According to the constructive episodic simulation hypothesis, episodic memory combines the details of past experiences (e.g. objects, people and locations) to depict future events that have not yet been experienced in the same form (Schacter and Addis 2007; Schacter et al. 2008). Notably, it has been demonstrated that individuals with organic episodic memory deficits from brain injuries present with difficulties in imagining future events (Addis et al. 2009; D’Argembeau et al. 2008; Hassabis et al. 2007). The heavy drinkers did not show episodic memory deficits on the prose recall task suggesting that when sober, they could encode and retrieve new information as efficiently as controls. Although they would experience episodic memory deficits when intoxicated, they would not show the consistent deficits over time associated with organic impairment. This would also fit with the finding that both groups in this study rated similar levels of imagery experience as each other, a finding replicating that of Griffiths et al. (2012).

### Methodological limitations

The sensitivity of virtual week to change with future event stimulation may have been improved by including more virtual days. This may help remove the apparent ceiling effect in the event-based PM performance of the control, social drinkers in both Griffiths et al.’s and the present study. Similar to other studies of the effects of an individual’s alcohol use, apart from ensuring no recent alcohol use by breathalysing, this study relied on self-report data of alcohol use history. Along with all self-report measures, these are susceptible to confound by acquiescence and/or social desirability. To remove any carry-over effects of future event simulation to the control condition, the control condition always preceded the experimental condition of future event simulation. At the expense of counterbalancing the order of the conditions, it remains unclear the extent to which future event simulation or practice effects or proactive interference effects account for the heavy drinkers and control groups’ improvements in PM. Although other drug use was relatively low across our sample, it is feasible that other drug use had impacted upon PM independent of alcohol use.

#### Clinical implications

Forgetting to carry out a planned action in the future has a wide range of implications in everyday life. For example, the financial cost of missed appointments in the UK National Health Service has been estimated to cost £360 million per year (Stone et al. 1999). Previous research has shown people with substance misuse to be significantly more likely than others to miss their NHS appointments (Sparr et al. 1993), and forgetting to be the most common reason for people missing follow-up appointments (Killaspy et al. 2000). The present results have important implications for heavy drinkers. Given their apparent deficits in PM abilities, the introduction of future event simulation may improve their attendance and subsequent desired outcomes from common treatments. Opportunities exist for health care professionals to routinely assess peoples’ alcohol consumption and offer brief interventions for hazardous levels of drinking. Due to the minimal time taken to provide instructions for future event simulation and the simple training required, this technique promises to be an effective adjunct to brief interventions for alcohol use. This may also go some way to limit individuals’ progression to alcohol-related pathologies, as well as the damage to physical and mental health associated with heavy drinking.

In summary, the present findings provide the first empirical evidence of prospective memory deficits for event-based tasks in treatment naïve, heavy drinkers and of the usefulness of future event simulation to overcome these deficits. Future research is needed to delineate and replicate the apparent effects of future episodic thinking. Prospective cohort studies may also go some way to elucidate dose-response relationships between alcohol and PM deficits.
